# Supplementary material for: Centrality measures in psychological networks: A simulation study on identifying effective treatment targets
Source: PLoS One. 2024 Feb 29;19(2):e0297058. doi: 10.1371/journal.pone.0297058 (PMC10903921; doi:10.1371/journal.pone.0297058)
Supplement: S1 Appendix — Network Basic Features. Graphical representation of the comparison between a degree-based normal attack and a degree-based cascade attack for the number of components and the average path length. Graphical representation of the comparison between a degree-based normal attack and a degree-based cascade attack for network density with 50% of the nodes removed. Plots Displaying Attack Results for Each Individual Network. (DOCX) [file pone.0297058.s001.docx]

**Centrality Measures in Psychological Networks: A Simulation Study on Identifying Effective Treatment Targets**

Supplementary Materials

Daniel Castro^1,2^, Deisy Gysi^3^, Filipa Ferreira^1,2^, Fernando Ferreira-Santos^4^, Tiago Bento Ferreira^1,2^

^1^University of Maia, Maia, Portugal

^2^Center for Psychology at University of Porto, Porto, Portugal

^3^Center for Complex Network Research, Northeastern University, Boston, Massachusetts, USA

^4^Laboratory of Neuropsychophysiology, Faculty of Psychology and Education Sciences, University of Porto, Portugal

Index

1. Table S1. Participants Characteristics
2. Table S2. Network Basic Features
3. Fig. S1 and S2. Comparison between Degree Cascade Attack and Degree Normal Attack
4. Fig. s3 – S83. Plots Displaying Attack Results for Each Individual Network
1. Table S1. Participants Characteristics

| **Network** | **Sex** | **Age** | **Ethnicity** | **Primary Diagnosis** | **Comorbidities** | **HAM-D** | **HAM-A** |
| --- | --- | --- | --- | --- | --- | --- | --- |
| 1 | Female | 18 - 24 | Latino | MDD | YES | 23 | 27 |
| 2 | Male | 25 - 30 | White | MDD | YES | 16 | 15 |
| 3 | Female | 25 - 30 | Latino | GAD | NO | 16 | 33 |
| 4 | Male | 18 - 24 | White | MDD | YES | 13 | 13 |
| 5 | Female | 25 - 30 | Black | MDD | YES | 11 | 17 |
| 6 | Female | 18 - 24 | Asian American | MDD | YES | 19 | 15 |
| 7 | Female | 18 - 24 | Other | MDD | YES | 17 | 9 |
| 8 | Male | 25 - 30 | Asian American | GAD | YES | 22 | 22 |
| 9 | Female | 31 - 40 | Latino | GAD | YES | 9 | 13 |
| 10 | Male | 18 - 24 | White | GAD | YES | 14 | 19 |
| 11 | Male | 18 – 24 | Latino | GAD | NO | 10 | 12 |
| 12 | Female | 25 – 30 | Asian American | MDD | YES | 10 | 10 |
| 13 | Male | 51 – 60 | Other | MDD | YES | 15 | 16 |
| 14 | Female | 51 – 60 | White | GAD | NO | 8 | 7 |
| 15 | Male | 25 – 30 | White | GAD | YES | 15 | 14 |
| 16 | Female | 18 - 24 | White | GAD | YES | 8 | 14 |
| 17 | Female | 18 – 24 | Latino | GAD | YES | 12 | 23 |
| 18 | Female | 25 – 30 | White | GAD | YES | 21 | 41 |
| 19 | Male | 51 – 60 | Asian American | GAD | YES | 14 | 17 |
| 20 | Female | 31 – 40 | White | MDD | NO | 11 | 14 |
| 21 | Female | 31 – 40 | Asian American | GAD | YES | 15 | 13 |
| 22 | Female | 51 – 60 | White | MDD | NO | 12 | 10 |
| 23 | Female | 18 – 24 | White | MDD | NO | 18 | 23 |
| 24 | Male | 25 – 30 | White | GAD | YES | 7 | 14 |
| 25 | Female | 18 – 24 | Asian American | GAD | YES | 18 | 15 |
| 26 | Female | 41 – 50 | Black | GAD | YES | 4 | 15 |
| 27 | Female | 31 – 40 | White | GAD | YES | 18 | 19 |
| 28 | Male | 51 – 60 | White | MDD | YES | 12 | 18 |
| 29 | Male | 25 – 30 | Latino | GAD | YES | 9 | 13 |
| 30 | Male | 41 – 50 | Asian American | MDD | NO | 16 | 15 |
| 31 | Female | 51 – 60 | White | MDD | YES | 14 | 12 |
| 32 | Female | 41 – 50 | Other | GAD | YES | 21 | 30 |
| 33 | Male | 41 – 50 | White | GAD | YES | 13 | 11 |
| 34 | Female | 51 – 60 | Asian American | MDD | YES | 16 | 16 |
| 35 | Male | 25 – 30 | White | MDD | NO | 13 | 15 |
| 36 | Female | 31 – 40 | White | GAD | YES | 10 | 11 |
| 37 | Female | 18 – 24 | Asian American | GAD | YES | 18 | 20 |
| 38 | Female | 51 – 60 | White | GAD | NO | 12 | 16 |
| 39 | Female | 31 - 40 | Black | GAD | NO | 17 | 23 |
| 40 | Female | 25 - 30 | White | GAD | YES | 17 | 14 |

2. Table S2. Network Basic Features

| **Network Characteristics** | **Networks (n=40)** | | |
| --- | --- | --- | --- |
|  | **Mean (SD)** | **Median (MAD)** | **Minimum-maximum** |
| Nodes | 21 | 21 (0.00) |  |
| Edges | 96.08 (15.42) | 101.00 (10.38) | 61 - 124 |
| Density | 0.46 (0.07) | 0.48 (0.05) | 0.29 - 0.59 |
| Components | 1 | 1 (0.00) |  |
| Average Path Length | 1.56 (0.11) | 1.52 (0.05) | 1.41 - 1.91 |

3. Comparison between Degree Cascade Attack and Degree Normal Attack

Figure S1. Graphical representation of the comparison between a degree-based normal attack and a degree-based cascade attack for the number of components and the average path length. In each panel, the result of the Wilcoxon signed-rank test for differences between the two attack conditions is presented. Boxplots represent the network density results range for each attack condition.

Figure S2. Graphical representation of the comparison between a degree-based normal attack and a degree-based cascade attack for network density with 50% of the nodes removed. In each panel, the result of the Wilcoxon signed-rank test for differences between the two attack conditions is presented. Boxplots represent the network density results range for each attack condition

5. Plots Displaying Attack Results for Each Individual Network

Plots displaying normal attack results for network 1

Plots displaying cascade attack results for network 1

Plots displaying normal attack results for network 2

Plots displaying cascade attack results for network 2

Plots displaying normal attack results for network 3

Plots displaying cascade attack results for network 3

Plots displaying normal attack results for network 4

Plots displaying cascade attack results for network 4

Plots displaying normal attack results for network 5

Plots displaying cascade attack results for network 5

Plots displaying normal attack results for network 6

Plots displaying cascade attack results for network 6

Plots displaying normal attack results for network 7

Plots displaying cascade attack results for network 7

Plots displaying normal attack results for network 8

Plots displaying cascade attack results for network 8

Plots displaying normal attack results for network 9

Plots displaying cascade attack results for network 9

Plots displaying normal attack results for network 10

Plots displaying cascade attack results for network 10

Plots displaying normal attack results for network 11

Plots displaying cascade attack results for network 11

Plots displaying normal attack results for network 12

Plots displaying cascade attack results for network 12

Plots displaying normal attack results for network 13

Plots displaying cascade attack results for network 13

Plots displaying normal attack results for network 14

Plots displaying cascade attack results for network 14

Plots displaying normal attack results for network 15

Plots displaying cascade attack results for network 15

Plots displaying normal attack results for network 16

Plots displaying cascade attack results for network 16

Plots displaying normal attack results for network 17

Plots displaying cascade attack results for network 17

Plots displaying normal attack results for network 18

Plots displaying cascade attack results for network 18

Plots displaying normal attack results for network 19

Plots displaying cascade attack results for network 19

Plots displaying normal attack results for network 20

Plots displaying cascade attack results for network 20

Plots displaying normal attack results for network 21

Plots displaying cascade attack results for network 21

Plots displaying normal attack results for network 22

Plots displaying cascade attack results for network 22

Plots displaying normal attack results for network 23

Plots displaying cascade attack results for network 23

Plots displaying normal attack results for network 24

Plots displaying cascade attack results for network 24

Plots displaying normal attack results for network 25

Plots displaying cascade attack results for network 25

Plots displaying normal attack results for network 26

Plots displaying cascade attack results for network 26

Plots displaying normal attack results for network 27

Plots displaying cascade attack results for network 27

Plots displaying normal attack results for network 28

Plots displaying cascade attack results for network 28

Plots displaying normal attack results for network 29

Plots displaying cascade attack results for network 29

Plots displaying normal attack results for network 30

Plots displaying cascade attack results for network 30

Plots displaying normal attack results for network 31

Plots displaying cascade attack results for network 31

Plots displaying normal attack results for network 32

Plots displaying cascade attack results for network 32

Plots displaying normal attack results for network 33

Plots displaying cascade attack results for network 33

Plots displaying normal attack results for network 34

Plots displaying cascade attack results for network 34

Plots displaying normal attack results for network 35

Plots displaying cascade attack results for network 35

Plots displaying normal attack results for network 36

Plots displaying cascade attack results for network 36

Plots displaying normal attack results for network 37

Plots displaying cascade attack results for network 37

Plots displaying normal attack results for network 38

Plots displaying cascade attack results for network 38

Plots displaying normal attack results for network 39

Plots displaying cascade attack results for network 39

Plots displaying normal attack results for network 40

Plots displaying cascade attack results for network 40
