# Supplementary material for: Centrality measures in psychological networks: A simulation study on identifying effective treatment targets
Source: PLoS One. 2024 Feb 29;19(2):e0297058. doi: 10.1371/journal.pone.0297058 (PMC10903921; doi:10.1371/journal.pone.0297058)
Supplement: Attachment — Submitted filename: Comments to Authors.docx [file pone.0297058.s002.docx]

**Comments to Authors**

This study utilizes a dataset accessible at [https://osf.io/mgdp6] and involves a sample of 40 participants, comprising 25 individuals with generalized anxiety disorder and 15 with major depressive disorder. The authors conducted a nuanced analysis of contemporaneous idiographic networks that characterize depression and anxiety symptoms within this cohort. Comparisons were made between cascade-based attacks and normal attacks. The authors suggest that degree centrality, as a more effective measure of network transformation, has considerable implications for future research aimed at delineating treatment goals through network analysis. Current evidence suggests that targeting symptoms through degree centrality-based cascading approaches may expedite more effective therapeutic interventions if changes in network structure correspond to psychopathology and health status.

The paper is overall well written and the message is clear. I have some minor comments as follows.

Overall

The author needs to define the first occurrence of the abbreviation. For example, "SD" in Method, Page 6; “LASSO” in Method, Page 7; “M” and “MAE” in Table.

Introduction, Page 4

What is “moderate support”? The description of “moderate” is a bit vague and difficult to understand.

Method

Did the authors consider the effects of sex and age differences in their study? Conducting sensitivity analyzes based on demographic variables such as sex or age can provide additional insights.If these factors were not included in the analysis, this may be a limitation of the study.

Table

The number of digits after the decimal point should remain consistent throughout the manuscript.

Figure

The figure legend appears to be incomplete, such as "degree, eigenvector, expected influence 1-step, and expected influence 2-step."
